# Cardiac Repair and Regeneration via Advanced Technology: Narrative Literature Review

**DOI:** 10.2196/65366

**Published:** 2025-03-08

**Authors:** Yugyung Lee, Sushil Shelke, Chi Lee

**Affiliations:** 1 Division of Pharmacology and Pharmaceutics Sciences School of Pharmacy University of Missouri Kansas City Kansas City, MO United States

**Keywords:** advanced technologies, genetics, biomaterials, bioengineering, medical devices, implantable devices, wearables, cardiovascular repair and regeneration, cardiac care, cardiovascular disease

## Abstract

**Background:**

Cardiovascular diseases (CVDs) are the leading cause of death globally, and almost one-half of all adults in the United States have at least one form of heart disease. This review focused on advanced technologies, genetic variables in CVD, and biomaterials used for organ-independent cardiovascular repair systems.

**Objective:**

A variety of implantable and wearable devices, including biosensor-equipped cardiovascular stents and biocompatible cardiac patches, have been developed and evaluated. The incorporation of those strategies will hold a bright future in the management of CVD in advanced clinical practice.

**Methods:**

This study employed widely used academic search systems, such as Google Scholar, PubMed, and Web of Science. Recent progress in diagnostic and treatment methods against CVD, as described in the content, are extensively examined. The innovative bioengineering, gene delivery, cell biology, and artificial intelligence–based technologies that will continuously revolutionize biomedical devices for cardiovascular repair and regeneration are also discussed. The novel, balanced, contemporary, query-based method adapted in this manuscript defined the extent to which an updated literature review could efficiently provide research on the evidence-based, comprehensive applicability of cardiovascular devices for clinical treatment against CVD.

**Results:**

Advanced technologies along with artificial intelligence–based telehealth will be essential to create efficient implantable biomedical devices, including cardiovascular stents. The proper statistical approaches along with results from clinical studies including model-based risk probability prediction from genetic and physiological variables are integral for monitoring and treatment of CVD risk.

**Conclusions:**

To overcome the current obstacles in cardiac repair and regeneration and achieve successful therapeutic applications, future interdisciplinary collaborative work is essential. Novel cardiovascular devices and their targeted treatments will accomplish enhanced health care delivery and improved therapeutic efficacy against CVD. As the review articles contain comprehensive sources for state-of-the-art evidence for clinicians, these high-quality reviews will serve as a first outline of the updated progress on cardiovascular devices before undertaking clinical studies.

## Introduction

Cardiovascular diseases (CVDs) are the leading cause of death globally, accounting for an estimated 17.9 million deaths in 2019 according to a report from the World Health Organization. Almost one-half of all adults in the United States have at least one form of heart disease [1]. Myocardial infarction (MI) is caused by ischemia in the coronary artery, primarily due to blocked arteries resulting from atherosclerosis [2]. This blockage damages the myocardium, reducing its contractile capacity, which leads to a decreased ejection fraction and, ultimately, heart failure [3]. In the United States, one healthy heart becomes infarcted every 40 seconds [4].

Preserving tissue and cellular function is crucial for maintaining heart functionality. Numerous signaling pathways and genetic factors associated with MI survival have been periodically reviewed [5-7]. There is a growing emphasis on understanding the mechanisms involved in myocardial repair and regeneration [8]. Reports from organizations such as the Transnational Alliance for Regenerative Therapies in Cardiovascular Syndromes highlight the importance of these mechanisms. Key principles affecting reparative and regenerative potential include survival and protection, cell-cell communication, angiogenesis and vascularization, cardiomyogenesis, molecular regulation of the cell cycle and proliferation, inflammation reduction, and cardiac aging [7,9].

An increase in reactive oxygen species (ROS) is a hallmark of ischemic cardiomyopathy [10]. ROS, such as hydrogen peroxide (H_2_O_2_) and hydroxyl radicals, play a significant role in MI and can be considered ideal regulators for patients post-MI [11]. The concentration of H_2_O_2_ in healthy cells is about 0.02 mM, whereas intracellular concentrations above 0.1 mM induce oxidative stress and cell death [12,13]. Given that extracellular H_2_O_2_ concentrations can be 10 to 100 times higher than intracellular levels [14], careful monitoring of H_2_O_2_ levels in cells is essential for prevention and treatment. As ROS play an integral role in platelet aggregation and vasodilation, inhibitors of vasodilation and platelet aggregation are commonly adapted as a therapeutic means against MI [15].

Regarding the treatment methods against CVD, organ transplant has been the most efficient strategy. Despite the preference for organ donor replacement in treating CVD, the shortage of organ donors has driven significant research into human-scale cardiovascular organs and functional tissue substitutes [16,17]. Challenges such as complex fabrication processes [18], poor mechanical properties [19], and biocompatibility and immunogenic issues [20] remain unresolved.

Designing prostheses requires fabricating matrix constructs with complex shapes and sizes for clinical applications [21]. Prostheses and implantable devices have varying requirements that are categorized into chemical, mechanical, electrical, and thermal characteristics [22]. Additionally, these devices must be biocompatible, be nonimmunogenic, and maintain functional capabilities within the body’s biological environment [23]. Although serious infections or side effects from cardiovascular prostheses are rare, infected prostheses can be fatal [24].

Hydrogels, which are hydrophilic polymeric scaffolds with unique 3-dimensional structures, can absorb large amounts of water or biological fluids, making them potential candidates for cardiovascular tissue engineering [23]. Various synthetic and natural polymers are used in implantable hydrogels, with natural polymers like collagen offering higher immunity and biodegradable properties over synthetic ones.

**Figure 1:**
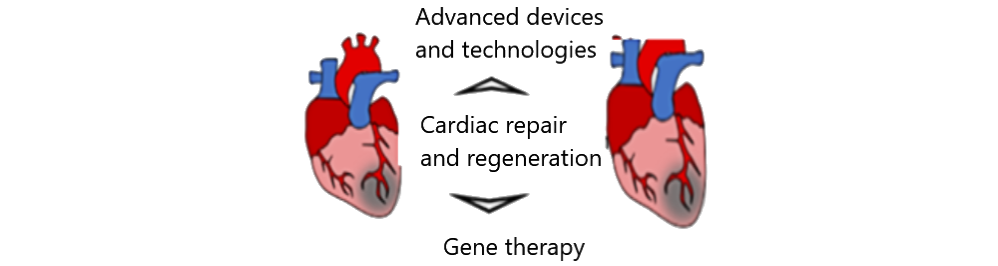
Cardiac repair and regeneration via advanced technologies and gene therapy.

This review focused on genetic variables in CVD, advanced technologies, and biomaterials for organ-independent cardiovascular repair systems (Figure 1). A variety of implantable and wearable devices, including biosensor-equipped cardiovascular stents and biocompatible cardiac patches, have been developed and evaluated. Finally, future research directions in the rapidly evolving fields of 3D-printed biomedical devices, artificial intelligence (AI), and multifunctional sensing devices are discussed.

## Advanced Assessment Technologies for Cardiac Image and Genetic Factors

### Image Features Extracted From Imaging Modalities

Risk variables used for the classification of CVD progression include radiological imaging features and genetic factors. The complex nature of cardiovascular structures makes stenosis assessment from image modalities a serious challenge. In general, imaging features are considered radiomic-based biomarkers or indicators rather than pathological symptoms. An assessment of imaging features can serve as a quantitative index extractable from such imaging modalities as magnetic resonance imaging, computed tomography angiography (CTA), and optical coherence tomography [25]. Even though a semiquantitative estimation of coronary stenosis is feasible via a thorough assessment of image features over an extended period, this process requires advanced technology expertise and labor-intensive effort.

In particular, coronary CTA, a noninvasive examination technique, plays an integral role in the evaluation and treatment of coronary artery disease (CAD) [26]. For instance, dual-source CTA allows for improved resolutions of implantable devices, including intrinsically higher-density stents, whose adversities are due to distortion reduction stemming from thick strut slices [27]. This approach makes it possible to conduct advanced cardiac imaging analysis, even though its invasive nature sometimes yields a high risk of fatality and complications [28-30].

As the number of images exponentially grows, the lack of ability to accurately label those images causes intrinsic limitations in the interpretation of the data [31]. A recent surge of AI techniques could serve as an ideal solution, enhancing the accuracy of a quantitative assessment of segmented features, including intima-media thickness ascertained by such computed algorithms as convolutional neural networks, UNet, UNet+, and DenseNet [32]. AI techniques and associated programmed models are for accurate identification of patterns, abnormalities, and defects in images, leading to enhanced efficiency and a reduction in errors inherent in human inspection [33].

### Evolving Gene Therapy Against CVD

#### Genetic Factors in the Assessment of CVD Risk

Genes are involved in most cardiovascular functions, starting with the robustness of blood vessels to the way cells interact. People with a family history of heart disease could share common environmental factors, such as the intake habits of drinking water and daily food and exposure to chemicals, including carbon monoxide, in the air. As most cardiac disorders, including arrhythmias, congenital heart disease, cardiomyopathy, and high blood cholesterol, can be inherited [34], assessing genetic variants or biomarkers to identify at-risk individuals is integral to the prevention and treatment of CVD [35].

Genetic variations acquired by children from parents in the DNA of the eggs and sperm can influence every cell of a child’s body, not only in the development process but also in the onset of heart disease [36]. An 8-year follow-up study found that CVD risk increased by 75% with a paternal history and about 60% with a maternal history of premature CVD, implying that certain genes can significantly enhance the risk of heart disease [37]. In the same study, a 16-year follow-up investigation found that a family history of premature CAD (age <50 years) marked a 44% higher risk of CVD mortality.

The pooled cohort equations for risk classification have been adapted based on genetic variants and medication decisions, including statins [38]. On the other hand, polygenic risk score (PRS) generation based on the relationships between the amount and frequency of genetic variants and the onset of specific diseases [39-41] has been explored for the assessment of genetic risk and extrapolation of individual outcomes [42]. The PRS could be accompanied by family history, lifestyle, and environmental factors [43,44] and fortified with emerging technologies, including proteomics, when determining an individual’s genetic predisposition to CVD [45,46]. PRS mostly outperforms traditional risk scores in the prediction of individual outcomes, and additional AI-based transfer learning could further upgrade the relatively less accurate performance on translating PRS from ancestry to different ethnicities that are mostly unknown and unvalidated [47].

Genes that could reduce the development of plaque around infected regions would prevent neointimal formation [48]. The primary CVD endogenous biological variants include C-reactive protein, a liver protein released in response to inflammation [8,49], and plasma levels of low-density lipoprotein cholesterol [50], a seminal risk factor for the development of coronary heart disease. In addition, pro-inflammatory CD4+ cells with CD28 expression [49,51], cardiac troponin I [52], and the number of regulatory T lymphocytes [53] are frequently examined as specific biomarkers for the diagnosis of acute MI. Also, specific genes (eg, APOB, LDLR, and PCSK9 genes for familial hypercholesterolemia and BAG3, LMNA, MYH7, PLN, RBM20, SCN5A, TTN, TNNC1, TNNI3, TNNT2, and TPM1 genes for dilated cardiomyopathy) were recommended by the American Heart Association to be tested for the diagnosis of monogenic CVDs [54].

Along with those biological variants, pathological genetic factors or symptoms assessed for CVD include carotid intima thickness [55,56] and vascular function (which occur in the early stage of familial hypercholesterolemia) [57,58]. Detection of those genetic markers as part of familial cascade screening programs in familial hypercholesterolemia can lead to preventive effects, where subsequent medical therapy can lower long-term CVD risk [55,59]. A combined application of various genetic factors based on each patient’s genetic profile may guarantee an efficient treatment strategy against CVD [35].

Even though genetic factors play a significant role in developing conditions of CVD, the screening processes including a health DNA test can only reveal certain genetic mutations that increase the risk and responses [60]. Subsequently, the relationship between genetic factors and risk scores is sometimes poor due to the fact that those having the genetic mutation do not necessarily have the same lifestyle factors, including basic health measures. Therefore, proper statistical approaches along with the results from clinical studies including model-based risk probability prediction from each or combined genetic variables are integral for genetic-based prediction of the CVD risk [61].

#### AI for Cardiovascular Gene Therapy

Genes (DNA, small interfering RNA, and microRNA) that could interfere with the development of plaque around infected regions are conjugated on biomedical devices like cardiovascular stents to prevent neointimal formation. An advanced monitoring process of genetic data and clinical data from electronic health records could lead to a fast and precise clinical decision and achieve customized treatment, eventually alleviating CVD via the detection of CVD symptoms at an early stage. However, the efficiency of cardiovascular gene therapy has been hampered by some obstacles, such as insufficient gene propagation, a lack of delivery mechanisms, and insufficient cell-vector interactions [62]. Moreover, health care providers may negatively influence clinical outcomes due to the lack of discipline in the treatment algorithms and the absence of established regulations to handle early-onset data [63,64].

Combined AI models will address highly complicated cardiovascular clinical genetics [65]. AI profoundly apprehends complex patterns in imaging profiles and offers quantitative assessments of radiographic properties, serving as a valuable tool for enhancing imaging postprocessing. For instance, a combined convolutional neural network and recurrent neural network has achieved enhanced accuracy in predicting stenosis (≥50%) upon examining genetic variables grouped into training and testing samples [32,66]. This approach has obtained similar outcomes in the quantitative assessment of the growing number of segmented image features, including intima-media thickness for CVD [31,32].

In general, the advanced technology involved with AI is revolutionizing the method that ensures the accuracy, completeness, consistency, and validity of clinically applicable gene data [67]. In parallel, researchers should follow established guidance on using information from the digital world, as several guidelines have already been issued by institutional review boards to properly maintain genetic data integrity [68]. As a result of the increase in genetic testing and the fear of privacy breaches by health providers, employers, and society, the disciplines of ethics, public health, and genetics have also emerged. The health professional should make a compromise between providing proper arrangements for patient care and protecting personal privacy. In the near future, the adaptation of AI in radiomics will lead to precise and automated analysis of genetic variables involved with disease onset and progress.

#### Telehealth Genetic Counseling Between Patients and Genetic Counselors

To improve the efficacy of the diagnosis and assessment of CVD risk, the prediction tools, including telehealth systems, should assess endogenous genetic compounds involved with heart failure, atherosclerosis, and CAD [67,69]. Telehealth genetic counseling, including videoconferencing and telephone counseling, was compared with in-person genetic counseling for the degree of outcomes specific to patient experiences and accessibility to various treatment methods. The patients expressed the highest satisfaction with genetic counseling provided by media devices, such as telephone and video [70-72]. Moreover, telehealth genetic counseling is considered equitable to in-person genetic counseling across numerous domains, even though those studies were conducted with telehealth systems that were less robust and accurate than what is available today.

The benefits and limitations of telehealth from the perspectives of the patients and genetic counselors have been thoroughly examined to resolve potential uncertainty in the analysis processes [73-75]. Those limitations include technical challenges, difficulty in rapport and the subsequent psychosocial issues, and lack of clinical complement [74,76]. There needs to be some conceptual changes in the current status of telehealth approaches over time, providing continuous advancement in involved technologies [76,77].

### Mobile Sensors for Cardiovascular Information Systems

**Figure 2:**
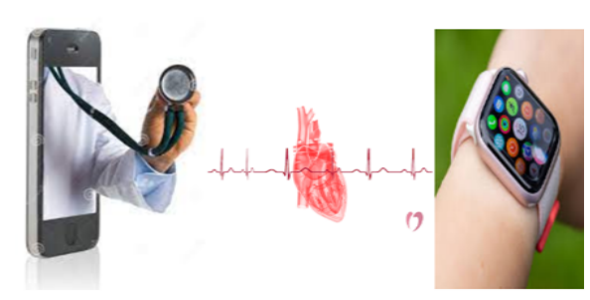
Schematic Representation of Remote Monitoring System of Biosensor/Cardiac Implantable Electronic Device.

Remote monitoring is considered the ambulatory tracing of vital signs and other medical indicators of a patient’s health and recovery status via a telemedicine system without the patient meeting doctors or being present in the clinic (Figure 2) [78]. The Food and Drug Administration has recognized the importance of devices such as continuous temperature monitoring or continuous glucose monitoring devices that allow health care providers to remotely monitor patients, including those that measure body temperature, respiratory rate, heart rate, and blood pressure. In addition, a new approach based on advanced technologies for various physiological variables and biomarkers has performed continuous in-time monitoring as well as subsequent customized treatment strategies.

The current roles of mobile sensors explored in telehealth technologies and further challenges in CVD will specifically emphasize (1) accurate assessment and diagnosis of vital signs or biomarkers from CVDs, (2) reliable and reproducible sensing systems to monitor the progress of a patient’s disease status, and (3) wearable devices with maintenance of battery life and restoration of interaction sensitivity capable of assessing cardiovascular information of patients at risk [79-81].

The problem arises when analyzing data from mobile sensors due to a lack of normalization and implementation of proprietary interfaces to the respective device or platform. In daily life, numerous portals provided by each sensor manufacturer should be simultaneously traced and aggregated into the existing database for each cardiovascular patient [82]. Thus, the integration of data obtained from patients with heart failure or implantable cardiac devices needs to be properly conducted to store data in a structured and interoperable way for timely clinical and scientific evaluations [83,84].

## Advanced Systems Currently Available for CVD

### Biomaterials for Organ-Independent Cardiovascular Repair Systems

#### Required Properties for Organ-Independent Cardiovascular Repair Systems

The highly ordered myocardium capacity for electrical integrity and electrical conduction between healthy and infarcted cells starts to diminish as the relatively disordered fibrous scar tissue disposition increases in the myocardium, leading to systolic and diastolic dysfunction and cardiac arrhythmia [85]. As heart transplantation is limited due to a shortage of organ donors, organ-independent systems, including cardiac patches, grafts, and scaffolds, play an essential role in cardiac repair and treatment of MI [86].

Biomaterial systems function like normal cardiac tissues, providing excellent electrical conductivity, mechanical strength, and biological activities to infarcted heart tissues [87]. Novel biomaterial-based systems offer self-renewal and regeneration in the damaged heart, serving as various resources for cardiac tissue repair for those with CVD. For instance, cardiac patches provide mechanical support to the myocardial wall and passively prevent the infarcted myocardium following MI by reducing myocardial wall stress and preventing left ventricular dilation and remodeling [88]**.**

#### Hydrogels for Organ-Independent Cardiovascular Repair Systems

Hydrogels are soft and moist injectable biomaterials with properties similar to those of human soft tissues. They are minimally invasive and serve as a vehicle for the delivery of therapeutic agents in situ [89,90]. Conductive hydrogel systems based on low-dimensional inorganic nanomaterials, such as carbon nanotubes and graphene derivatives [23], and simultaneously loaded with stem cells, growth factors, cytokines, or oligonucleotides, are found to alleviate cardiac casualties by promoting angiogenesis and cardiomyocyte proliferation and reducing fibrosis and apoptosis.

In addition, a complex hydrogel patch is produced by principles of fabrication via Fe^+3^-induced ionic coordination between a homogeneous network of dopamine-gelatin conjugates and dopamine-functionalized polypyrrole [91]. The Schiff base reaction between oxidized sodium hyaluronic acid and hydrazided hyaluronic acid was explored to form an injectable hydrogel patch. Added bioactive peptides, a 7-amino acid peptide, loaded in collagen-based hydrogel reduced cell apoptosis, enhanced Sca-1+ recruitment and differentiation of stem cells, and enhanced neovascularization formation, which resulted in improved heart function in a mouse MI model [90].

### Cardiac Patch

#### Therapeutic Patch as an Effective Strategy

All the delivery methods for MI recovery drugs, primarily via the oral route but occasionally via an intravenous route, direct injection to the heart, and drug-eluting stents, have their own limitations in resolving MI-induced loss of cardiomyocytes [92]. Advanced formulations, including cardiac patches, have demonstrated their efficiencies in functional recovery for drug carriers with targeted and local delivery of cardiovascular drugs, nutrients, and cells. Moreover, patches not only are capable of providing necessary mediators in multiple therapies to recover the affected area but also strengthen the damaged area with induced cell attachment and proliferation [93].

#### Types of Patches and Their Applications for MI Recovery

Therapeutic patches are divided into two types based on the presence or absence of cells: cell-based patches and acellular patches. As there is a lack of regeneration of cardiomyocytes, cells such as human-induced pluripotent stem cells, mesenchymal stem cells, and skeletal myoblasts are often introduced to restore cardiac function [94].

Newly introduced cells can lead to enhanced angiogenesis, lowered fibrosis, and apoptosis of cardiomyocytes [2]. Due to the inefficiency of generating new heart tissue from cardiomyocytes, acellular cardiac patches, which might include paracrine factors such as proteins, RNA, growth factors, or small molecules, are occasionally explored to accomplish cardioprotective effects [95].

The biocompatibility of the source biomaterial often entails a serious challenge in designing any implantable patches [96]. Moreover, the biomaterial should be similar to that of host tissues from the perspectives of biochemical, mechanical, and topographical properties [97,98]. For instance, poly(hydroxyethyl) methacrylate (pHEMA) polymer has demonstrated biocompatibility and has been used for biomedical applications, including drug delivery [99,100], contact lenses [101,102], and tissue engineering [103,104]. However, the low viscous nature of pHEMA makes it a challenging task to develop pHEMA-based biomedical devices, including a cardiac patch that is capable of successfully delivering agents like ROS scavengers against MI.

#### 3D Printing Technology for Cardiac Patch Development

3D printing can be used to create patient-specific devices, such as organ implants and tissue models that mimic human physiology. 3D printing can generate surgical planning models and reduce the need for animal testing. 3D printing can be used to create personalized medicines and their delivery systems that specifically adapt to each patient’s genetic makeup [105].

There are numerous methods, including electrospinning, solvent evaporation, and decellularization, used for the development of patches [106]. Each of these methods has its own challenges, such as material selectivity, limitations in complex shapes, and cost and time efficiency [107]. Additionally, 3D printing has emerged as a low-cost and fast method to develop patches produced from a vast range of materials with the utmost efficacy.

As previously mentioned, a novel patch based on biocompatible pHEMA polymers was developed with the aid of direct-light 3D printing technology. Stereolithography-based 3D printing, where the ink is placed on a platform, was successfully used to prepare 3D-printed acellular cardiac patches or cardiovascular stents [21]. In 3D-printed systems, the immunosuppressive drug, like sirolimus, dispersed within the patch matrix will be released when the linker, like an ROS-responsive thioketal linker, that connects the polymers is cleaved [108]. The ratio of the polymer and crosslinker can be customized to achieve controllable drug release.

### 3D- or 4D-Printable Smart Devices for CVD

3D printing provides geometric flexibility, which has been explored to produce metal or polymer-embedded 3D construct microsystems with high flexibility [23,109]. 3D-printed systems or smart devices use advanced materials with characteristics such as thermal and electrical conductivity and piezo-resistivity [110]. Electric units or components, including resistors, capacitors, inductors, circuits, and passive wireless sensors and batteries, have been incorporated into 3D-printed products for potential practical applications.

3D tactile sensors capable of detection and differentiation of human movements, including pulse monitoring and finger motions via detection of endogenous compounds, were fabricated using multimaterial, multiscale, and multifunctional approaches under ambient conditions conformally onto freeform surfaces [111]. As lactate levels have been associated with heart failure as well as diabetes, the portable luminometer, a disposable minicartridge produced by 3D printing and stored in cell phones, was used to detect chemiluminescence from enzyme-coupled reactions [112]. Lactate oxidase was coupled with horseradish peroxidase to noninvasively detect the lactate levels within 5 minutes at a detection limit of 0.5 mM/L and 0.1 mM/L in oral fluids and sweat, respectively.

By adapting AI to additive manufacturing, 3D designers can optimize cardiovascular biosensors or implants to be more efficient and robust. AI-mediated 3D printing tools can synchronize with high-quality imaging data, such as computed tomography and magnetic resonance imaging scans, and generate personalized designs, enabling thorough control over the otherwise unavoidably complicated, time-consuming, and exhaustive process [3].

An optimal combination of 3D printing based on novel or hybrid 3D printing methods and AI can achieve the next generation of cardiovascular systems [113]. Subsequently, advanced 3D or 4D printing, once nearly overcoming the cost and scalability barriers, could lead to more effective and targeted treatments against CVD, accomplishing improved treatment outcomes and enhanced health care delivery [67].

### Advanced Cardiovascular Stents for CVD

#### Gene-Eluting Stents

Advanced biomedical gene carriers have been intensively explored in vascular cell biology and CVD treatment. The identification of critical regulators, such as noncoding RNAs, including microRNA, long noncoding RNA, and circular RNA presence in such cell types as vascular smooth muscle cells, endothelial cells, and macrophages, has served as an efficient therapeutic target in the field of CVD.

Among biomedical carriers, multifunctional gene-loaded stents and integrated stents equipped with self-reporting sensors are often explored as promising technologies against CVD, including atherosclerosis and MI [114,115]. Cardiovascular stents keep the vessel open and prevent it from re-occluding (ie, restenosis), but vessel injury by stent struts leads to the activation of platelets and mural thrombus formation, leading to the activation of circulating neutrophils and tissue macrophages [116-118]. As the cardiovascular stent produces late-stage restenosis [119,120], people with stents are at risk of high blood pressure. Therefore, it is integral to find a more advanced and sensitive stent capable of real-time monitoring of blood flow.

Gene-loaded stents coated with synthetic and natural polymers such as polylactic-polyglycolic acid (PLGA), collagen, hydroxyapatite, and matrix metalloproteins can overcome major limitations of cardiovascular gene therapy, including insufficient cell-vector interactions, a lack of delivery mechanisms, and insufficient gene propagation [121]. Gene-loaded stents also allow for maintaining a curative gene, serving as a carrier to convey the gene and administer the vector and avoiding immune responses [62].

The first successful in vivo transfection of green fluorescent protein plasmid DNA loaded in a DNA-PLGA coated stent was efficiently expressed in cell cultures (7.9%, SD 0.7% vs 0.6%, SD 0.2% control; *P*<.001) of rat aortic smooth muscle cells [122]. In addition, PLGA nanoparticle-coated stents encapsulated with vascular endothelial growth factor and paclitaxel [123] or Ang-1 proteins [124] were developed as an alternative therapy, reducing in-stent restenosis and accomplishing complete re-endothelialization. In addition, an Akt1 small interfering RNA–embedded stent alleviated restenosis, reducing cell growth via muting RNA [125,126]. Furthermore, bare-metal stents with a synthetic complex for reversible vector binding produced prominent green fluorescent protein positivity in A10 cells proximal to the strut after 72 hours in culture [127].

A collagen-coated stent covalently coupled with anti-DNA immunoglobulin M antibody and loaded with plasmid DNA was efficiently developed for localized gene delivery to smooth muscle cells in an artery, accomplishing high-level protein production through reporter gene expression [125]. In addition, a stent coated with biomimetic hyaluronic acid and deposited with DNA/polyethylenimine polyplexes was explored to deliver plasmid DNA to the artery, exerting its efficacy in alleviating restenosis with a higher neointimal transfection rate while maintaining structural stability [128].

#### Stents Equipped With Cardiovascular Self-Reporting Sensors

Continuous blood flow surveillance can serve as screening, advanced detection, and alert for cardiovascular health using noninvasive technology that can be placed in the coronary arteries [129]. Remote monitoring of patient progress is feasible by creating an application-specific integrated circuit that features a voltage regulator and radio frequency power element loaded in biomedical devices, including cardiovascular stents.

For instance, a remote monitoring stent was combined with a tiny heart pressure sensor as well as a wireless transmitter that continuously monitors vascular conditions and the status of implanted devices. To minimize the number of antenna components for the conservation of space, the stent was used as an inductive antenna to create a wireless network [130,131], transmitting quantified solubilization to the immediate neighborhood via a wireless telemetry transmitter [132]. Reviewing the admittance of an antenna close to the implant component and connected to it via electromagnetic coupling will enable this function [133]. A radio frequency–powering component was implanted on the chip in the finished device as an ideal power distribution feature. Microelectromechanical modules were crammed with an application-specific integrated circuit for data collection [134].

**Figure 3:**
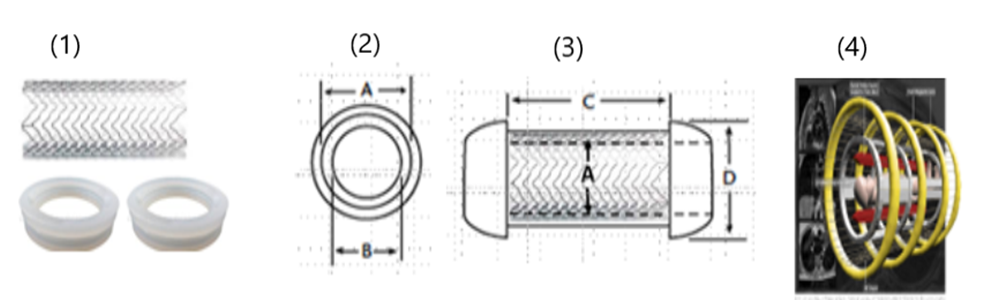
(1) stent and rings, (2) dimension and size of rings (A: Ring inner diameter (i.e., same as stent outer diameter); B: Stent inner diameter), (3) 3-Dimentional Stent (C: Stent length; D: Ring outer diameter) and (4) application of external electromagnetic stimuli.

As shown in Figure 3, a blood flow sensor enclosed in graphene-embedded silicon rings subsequently equipped with a digital wireless transmission microchip was developed as a unit of the smart theranostic cardiovascular stent (Figure 3C). Numerous commercial devices, including pressure sensors, use the piezoresistive effect of silicon, whose gauge factors can be 2 orders of magnitude larger than those observed in most metals [135,136]. Thus, a flow sensor enclosed in the stent was able to continuously monitor real-time blood flow with high inductance and pressure resolution and transmit corresponding data to a cardiologist outside the body. In addition to superior moisture barrier property, the high thermal conductivity of graphene (which has a negative thermal expansion coefficient [−8.0 × 10^–6^/K] between 0 and 700 K) guaranteed dimensional stability upon exposure to body temperature and continuous blood flow.

The pressure sensors and the microchip were mounted on the rectangular areas of the stent structure, as shown in Figure 3C. The pressure sensors bound to the steel stent [137,138] were molded into graphene-embedded silicone rings, and the pattern was cut on a thin stainless-steel foil. These digital transmission techniques reduced the power radiated by the external reader, thus minimizing the patient’s exposure to electromagnetic fields.

In electromagnetic coupling, a continuous electromagnetic wave with relatively large power is radiated by the reader, and the microchip modulates the impedance of the antenna by connecting or disconnecting a load to it according to the data to be transmitted [130].

## Conclusion

Biotechnologies play an important role in cardiovascular repair and regeneration. Genetic variables in CVD, currently available technology, and biomaterials for organ-independent cardiovascular repair systems were updated in this article in a timely manner. Advanced biotechnologies aimed at target-specific therapeutics are designed for customized and personalized cardiac treatment strategies with one or multiple administration routes whose methods should be further improved to enhance targeting and treatment efficacy.

The goal of gene therapy for cardiac repair and regeneration is to achieve cardiac transfection outcomes via the selection of proper gene vectors and modifying a gene or genetic pathways. Moreover, 3D bioprinting technology has been widely used in cardiac repair by integrating biomaterials with various manufacturing processes to customize cardiac conditions. 3D scaffolds with varying cell types have demonstrated better biocompatibility, delivery efficiency, and low immunogenicity. In the future, screening and designing of viral vectors through structure evolution mediated by 3D printing would enhance cardiac gene therapy.

To overcome the current obstacles in cardiac repair and regeneration and achieve successful therapeutic applications, future interdisciplinary collaborative work should be integral. Advanced new material and cell biology, along with AI-based telehealth, will be essential to create efficient implantable biomedical devices, including cardiovascular stents. Advanced innovative bioengineering, gene delivery, and cell biology technologies will continuously revolutionize medical devices for cardiovascular repair and regeneration in the future.

## Abbreviations

AI: artificial intelligence
CAD: coronary artery disease
CTA: computed tomography angiography
CVD: cardiovascular disease
H2O2: hydrogen peroxide
MI: myocardial infarction
pHEMA: poly(hydroxyethyl) methacrylate
PLGA: polylactic-polyglycolic acid
PRS: polygenic risk score
ROS: reactive oxygen species
